# Obesity is associated with insulin resistance and components of the metabolic syndrome in Lebanese adolescents

**DOI:** 10.3109/03014460.2012.655776

**Published:** 2012-02-13

**Authors:** Lara Nasreddine, Farah Naja, Maya Tabet, Mohammad-Zuheir Habbal, Aida El-Aily, Chrystel Haikal, Samira Sidani, Nada Adra, Nahla Hwalla

**Affiliations:** 1 Department of Nutrition and Food Science, Faculty of Agricultural and Food Sciences, Faculty of Medicine, American University of Beirut, Lebanon; 2 Department of Pathology and Laboratory Medicine, Faculty of Medicine, American University of Beirut, Lebanon

**Keywords:** Metabolic syndrome, obesity, body fat, BMI, waist circumference, insulin resistance, adolescents, Lebanon

## Abstract

**Background:**

Prevalence of metabolic syndrome (MS) in obese adolescents has been reported to range between 18–42%, depending on country of origin, thus suggesting an ethnic-based association between obesity and MS.

**Aim:**

This study aims to investigate the magnitude of the association between obesity, insulin resistance and components of MS among adolescents in Lebanon.

**Subjects and methods:**

The sample included 263 adolescents at 4^th^ and 5^th^ Tanner stages of puberty (104 obese; 78 overweight; 81 normal weight). Anthropometric, biochemical and blood pressure measurements were performed. Body fat was assessed using dual-energy X-ray absorptiometry.

**Results:**

According to International Diabetes Federation criteria, MS was identified in 21.2% of obese, 3.8% of overweight and 1.2% of normal weight subjects. The most common metabolic abnormalities among subjects having MS were elevated waist circumference (96.2%), low HDL (96.2%) and hypertriglyceridemia (73.1%). Insulin resistance was identified in all subjects having MS. Regression analyses showed that percentage body fat, waist circumference and BMI were similar in their ability to predict the MS in this age group.

**Conclusions:**

MS was identified in a substantial proportion of Lebanese obese adolescents, thus highlighting the importance of early screening for obesity-associated metabolic abnormalities and of developing successful multi-component interventions addressing adolescent obesity.

## BACKGROUND

The prevalence of paediatric obesity has increased over the last decades in many countries, with estimates indicating higher obesity prevalence among adolescents in the Eastern Mediterranean Region (EMRO) than that reported from Europe and the US (Kelishadi 2007). The increase in the prevalence of obesity in youths has been parallelled by an increase in the prevalence of paediatric metabolic syndrome (MS), a constellation of metabolic risk factors that include insulin resistance, hypertension, glucose intolerance and an abnormal lipid profile (Weiss et al. 2004; Esmaillzadeh et al. 2006; Braga-Tavares and Fonseca 2010). A recent study conducted in Turkey showed that, among 2–19 year old children and adolescents, a one-point increase in BMI *Z*-score resulted in a 2-fold increase in the prevalence of MS (Sen et al. 2008).

Based on the modified ATPIII criteria (Cook et al. 2003), prevalence estimates of MS among obese adolescents were found to vary between 18% in Spain and 42% in the US, suggesting an ethnic-based association between obesity and MS (López-Capapé et al. 2006; Dhuper et al. 2007). Limited data from the Middle-East suggest high rates of MS and cardiometabolic risk factors in the adolescent population. The prevalence of MS among obese adolescents in Iran is among the highest estimates reported in the literature (41.9%) (Esmaillzadeh et al. 2006). Similarly, in a recent study conducted on 203 overweight and obese subjects aged 6–17 years living in Norway, the prevalence of MS was found to be higher among obese subjects with Middle Eastern origins than among their Norwegian counterparts (30.6% vs 20.8%) (Kolsgaard et al. 2008). This association between adolescent obesity and MS indicators has not been investigated in many countries of the Middle-East.

Although recent definitions of MS do not include direct measurement of insulin in children (Eyzaguirre and Mericq 2009), insulin resistance (IR) has been frequently reported to overlap with MS and to confer cardiometabolic disease risk distinct from that attributed to MS itself (Haffner et al. 1990; Dhuper et al. 2007; Steinberger et al. 2009). Ethnic disparities in insulin resistance have also been reported in the literature, with insulin resistance suggested as being more frequent in White and African-American people and less common in Asiatic, Arabic and Latin American populations (Caceres et al. 2008).

Even though the clinical utility of MS as a disease category faces considerable controversy, particularly when applied to the paediatric population, the screening for MS and its components early in life allows for the identification of individuals at significant risk for cardiovascular disease and those in urgent need of lifestyle intervention. Recent evidence suggests the presence of early functional and morphologic changes to the heart and blood vessels among obese adolescents with MS (Chinali et al. 2008). Yet, the plasticity of the cardiovascular system early in life allows for the reversal of cardiovascular damage and cardiac abnormalities in obese adolescents, but only if risks are identified early and treated aggressively (Ippisch et al. 2008; Battista et al. 2009). For these reasons, the identification of paediatric MS in individuals who have not yet developed cardiovascular disease, is of great importance from a public health perspective.

This study was conducted to investigate the magnitude of the association between obesity, insulin resistance and various components of the MS among adolescents (Tanner stages 4–5) in Lebanon, a small Eastern Mediterranean country, where prevalence rates of paediatric obesity are high, approaching those observed in developed countries such as the US (Sibai et al. 2003).Confirming MS prevalence in Lebanese obese adolescents should lead to multi-component interventions targeting MS and its components in Lebanon and other parts of the Middle-East.

## METHODS

### Study design and participants

This is a cross-sectional study of adolescents attending public schools in Beirut. The research was approved by the Institutional Review Board, American University of Beirut.

A sample of 263 subjects was recruited using a multistage cluster sampling as follows. Out of 74 public schools in Beirut, eight were randomly selected from the list of the Lebanese Ministry of Education. From grades 10, 11 and 12, a BMI-stratified sample was conveniently recruited. Stratification was based on BMI status as defined by the new WHO growth standards (de Onis et al. 2007). Accordingly, obesity was defined as BMI > +2 *Z*-scores for sex and age, overweight as + 1 < BMI *Z*-scores ≤ + 2 and normal weight as BMI ≤ + 1 BMI *Z*-scores (de Onis et al. 2007). Written informed consent from parents and written informed assent from participants was sought. Subjects were invited to visit the research centre at the Nutrition and Food Science Department at the American University of Beirut and were instructed to consume a daily diet providing 50–60% of energy intake as carbohydrates for 3 days prior to their visit and to avoid any vigorous physical activity. Eligibility of study subjects was verified. Subjects were eligible if they were healthy, at 4^th^ and 5^th^ Tanner stages of puberty, as determined by a medical doctor, and of Lebanese ancestry. Exclusion criteria included history of chronic illness or the use of medications that alter blood pressure, glucose or lipid metabolism.

Sample size calculation was based on the following: To detect a moderate correlation (*r*) of a range of 0.2–0.3 between obesity and components of the metabolic syndrome, a sample of 68–150 subjects was needed to provide an 80% power at *p* = 0.05.

### Data collection

Data collection was carried out in 2008 – 2009 on participants who matched inclusion criteria and had provided written assent as well as parental written informed consent. Anthropometric measurements were taken after voiding and included weight, height, WC and %BF which was measured by dual-energy X-ray absorptiometry (DXA) (Delphi W, Hologic Inc, QDR software for windows version 11.2) based on region %BF. Waist circumference (WC) was measured using a calibrated plastic measuring tape, at the umbilicus level, with subjects standing and following normal expiration (Marfell-Jones et al. 2006). Fasting blood samples (10 ml/participant) were obtained by a phlebotomist for the analysis of serum glucose, insulin, triglycerides (TG) and HDL. Blood pressure was measured by a registered nurse using a mercury sphygmomanometer, with the subjects seated and after a 5-minute rest.

### Biochemical analysis

Plasma glucose was analysed using the COBAS 6000 analyzer (Roche Diagnostics, Switzerland). Serum insulin was determined by the use of an antibody radioimmunoassay kit (Linco Research, Missouri, USA). Serum TG was determined using the COBAS 6000 analyzer (Roche Diagnostics, Switzerland) while serum HDL was colourimetrically determined by the use of the HDL separation tab (Union Carbide Corp. Pleasantville, NY, USA).

### Diagnostic criteria for the metabolic syndrome

MS was defined based on the recently published harmonized metabolic syndrome definition (Alberti et al. 2009), according to which three abnormal findings out of five would qualify the subject for the MS. The cut-off values for the individual metabolic abnormalities are based on the IDF criteria (Zimmet et al. 2007), which recommend the use of the adult IDF cut-off values for subjects aged 16 years and older, as follows: abdominal obesity (WC s 94 cm for males and s 80 cm for females); elevated TG (≥ 150 mg/dL); low levels of HDL (< 40 mg/dL for males and < 50 mg/dL for females); high systolic blood pressure (SBP) (≥ 130 mmHg) or high diastolic blood pressure (DBP) (≥ 85 mmHg); high fasting glucose (≥ 100 mg/dL). For subjects aged 10–16 years, the IDF recommends the use of the same diagnostic criteria except for WC and HDL, where WC ≥ 90^th^ percentile for age and sex, and HDL < 40 mg/dL were used as cut-off values. For comparative purposes, the data were also analysed according to the modified ATP III definition (Cook et al. 2003).

As reference values specific for adolescents in Lebanon are not available, hyperinsulinemia was determined based on cut-off points specific for the Tanner stage of maturation (fasting insulin ≥ 30 mlU/L for Tanner stage 4; fasting insulin ≥ 20 mlU/L for Tanner stage 5) proposed by the literature (Goran and Gower 2001; Sen et al. 2008). Insulin resistance (IR) was determined using the homeostatic model assessment of insulin resistance (HOMA-IR) defined as:

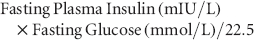
 Values exceeding 3.16 indicated the presence of IR (Keskin et al. 2005).

### Statistical analysis

Statistical analysis was performed using the Statistical Analysis Package for Social Sciences (SPSS, version 16.0) and the level of significance was set at *p* < 0.05. Frequencies and descriptive statistics were performed for the different variables under study including MS prevalence according to different definitions as well as the prevalence of individual components of MS. Means and standard deviations were computed for anthropometric and biochemical variables. Mean differences were compared for significance between groups, using one-way ANOVA. Pearson correlations were performed between obesity indicators and the different MS components. The association between MS abnormalities (dependant variables) and adiposity indicators including %BF, WC and BMI (independent variables) was assessed by age and sex- adjusted linear regression analyses. Natural logarithm transformation was performed when the dependent variable was not normally distributed. Determinants of MS were evaluated using age- and sex-adjusted logistic regression analysis.

## RESULTS

The study sample consisted of 263 adolescents (112 males and 151 females) and included 104 obese, 78 overweight and 81 normal weight subjects. Anthropometric, biochemical and blood pressure measurements of the study subjects are shown in Table I. Genders combined, mean serum TC, LDL-C, TG, insulin levels, systolic and diastolic blood pressure were significantly higher and HDL-C significantly lower in obese as compared to normal weight adolescents. The most common abnormality among obese adolescents was elevated WC (90.4%), followed by IR (88.5%) and hyperinsulinemia (73.1%), while hyperglycemia was the least common abnormality (3.8%) (Table II).

**Table I:** Baseline characteristics of the adolescent subjects.

	Adolescents		
	
Characteristics	Obese > + 2 BMI *z*-scores (*n* = 104)	Overweight + 1 < BMI *z*-scores ≥ + 2 (*n* = 78)	Normal weight ≤ + 1 BMI *z*-scores (*n* = 81)
Age (years), mean ± SD	16.07 ± 1.30	16.39 ± 1.44	16.80 ± 1.27
Anthropometric data, mean ± SD			
BMI (Kg/m^2^)	32.52 ± 3.39^a^	26.38 ± 1.35^b^	20.87 ± 1.98^c^
Body Fat (DXA) (%)	38.86 ± 5.65^a^	32.95 ± 7.29^b^	23.60 ± 8.76^c^
WC (cm)	97.86 ± 11.57^a^	85.16 ± 8.95^b^	73.06 ± 8.38^c^
Biochemical and blood pressure data, mean ± SD			
TC (mg/dL)	156.78 ± 27.34^a^	154.19 ± 30.44^a^	141.63 ± 21.92^b^
HDL (mg/dL)	43.66 ± 10.52^a^	46.80 ± 10.33^b^	48.10 ± 9.98^b^
LDL (mg/dL)	93.84 ± 25.74^a^	90.60 ± 26.72^a^	79.86 ± 18.69^b^
TG (mg/dL)	105.09 ± 50.39^a^	85.58 ± 42.76^b^	68.31 ± 26.05^c^
FBG (mg/dL)	88.62 ± 8.10^a^	88.62 ± 7.11^a^	89.46 ± 5.48^a^
Insulin (μU/mL)	33.55 ± 18.37^a^	26.42 ± 14.38^b^	10.64 ± 7.89^c^
SBP (mmHg)	117.38 ± 11.97^a^	113.08 ± 9.98^b^	113.65 ± 10.41^b^
DBP (mmHg)	70.67 ± 8.16^a^	66.99 ± 6.95^b^	65.33 ± 5.44^b^

a Values in the same row with different superscripts are significantly different *p* < 0.05 (one-way ANOVA between normal weight vs overweight vs obese groups).

b Values in the same row with different superscripts are significantly different *p* < 0.05 (one-way ANOVA between normal weight vs overweight vs obese groups).

c Values in the same row with different superscripts are significantly different *p* < 0.05 (one-way ANOVA between normal weight vs overweight vs obese groups).

The definitions of overweight and obesity were based on sex- and age-specific +1 and +2 BMI *z*-scores, respectively, derived from WHO child growth standards (de Onis et al. 2007).

DBP, Diastolic Blood Pressure; FBG, Fasting Blood Glucose; HDL-C, High Density Lipoprotein-Cholesterol; SBP, Systolic Blood Pressure; TC, Total Cholesterol; TG, Triglycerides; WC, waist circumference.

**Table II:** Prevalence of individual metabolic abnormalities by BMI classification.

	Adolescents, *n* (%)		
	
Individual abnormalities	Obese > + 2 BMI *z*-scores (*n* = 104)	Overweight + 1 < BMI *z*-scores ≤ + 2 (*n* = 78)	Normal weight ≤ + 1 BMI *z*-scores (*n* = 81)
Elevated Waist Circumference^a^	94 (90.4)	36 (46.2)	3 (3.7)
Low HDL^a^	45 (43.3)	29 (37.2)	27 (33.3)
High tryglycerides^a^	21 (20.2)	6 (7.7)	1 (1.2)
Hypertension^a^	18 (17.3)	5 (6.4)	9(11.1)
High fasting glucose^a^	4 (3.8)	6 (7.7)	3 (3.7)
Insulin resistance^b^	92 (88.5)	66 (83.3)	20 (24.7)
Hyperinsulinemia^c^	76 (73.1)	45 (57.7)	7 (8.6)

a As defined by IDF Criteria:

• Elevated Waist Circumference (WC): For subjects 16 years and older, WC ≥ 94cm for males and ≥ 80 cm for females. For ages 13–15 years, WC ≥ 90th percentiles or adult cut-off value if lower;

• Low HDL: For subjects 16 years and older HDL < 40 mg/dL for males and < 50 mg/dL for females. For ages 13–15 years, HDL < 40 mg/dL;

• High TG: TG ≥ 150 mg/dL;

• Hypertension: SBP ≥ 130 mmHg or DBP ≥ 85 mmHg; and

• High Fasting Glucose: ≥ 100 mg/dL.

b Insulin resistance, HOMA-IR > 3.16;

c Hyperinsulinemia, Insulin ≥ 30 μU/ml for Tanner stage 4 and ≥ 20 μU/ml for Tanner stage 5.

The number of metabolic abnormalities increased with obesity (Table III), whereby 19.2% of obese subjects had three metabolic abnormalities as compared to 3.8% of overweight subjects and 1.2% of the normal weight subjects. The presence of four metabolic abnormalities was only noted among obese subjects (1.9%). Based on IDF criteria, the prevalence of MS was 21.2% in obese subjects, 3.8% in overweight subjects and 1.2% in the normal weight subjects. Analysing the data according to the modified ATP III definition (Cook et al. 2003) yielded a slightly higher prevalence of MS in the obese study sample (24.0%), while MS prevalence among overweight and normal weight subjects remained essentially the same (3.8% in overweight and 1.2% in normal weight subjects). Gender disparities were noted with both the IDF (27.1% in obese boys and 16.1% in obese girls) and ATPIII (31.3% in obese boys and 17.9% in obese girls) definitions, with obese boys showing higher prevalence rates of MS.

**Table III:** Number of metabolic syndrome abnormalities, according to IDF 2009 criteria.

	Adolescents, *n* (%)		
	
Metabolic syndrome abnormalities	Obese > +2 BMI *z*-scores (*n* = 104)	Overweight + 1 < BMI *z*-scores ≤ + 2 (*n* = 78)	Normal weight ≤ + 1 BMI *z*-scores (*n* = 81)
One	44 (42.3)	33 (42.3)	32 (39.5)
Two	35 (33.7)	20 (25.6)	4 (4.9)
Three	20 (19.2)	3 (3.8)	1 (1.2)
Four	2 (1.9)	0 (0.0)	0 (0.0)
Metabolic syndrome	22 (21.2)	3 (3.8)	1 (1.2)

Among subjects having the MS, the most common metabolic abnormalities as defined by the IDF were elevated waist circumference (96.2%) and low HDL (96.2%) followed by hypertriglyceridemia (73.1%), while the least common metabolic abnormality was hyperglycaemia (7.7%) (Table IV).

**Table IV:** Individual abnormalities among subjects with metabolic syndrome (*n* = 26), according to IDF (2009).

Individual abnormalities	Subjects with the metabolic syndrome, *n* (%)
Elevated WC^a^	25 (96.2)
Low HDL^a^	25 (96.2)
High TG^a^	19 (73.1)
Hypertension^a^	9 (34.6)
High fasting glucose^a^	2 (7.7)
Insulin resistance^b^	26 (100.0)
Hyperinsulinemia^c^	19 (73.1)

a As defined by IDF Criteria:

• Elevated Waist Circumference (WC): For subjects 16 years and older, WC ≥ 94 cm for males and ≥ 80 cm for females. For ages 13–15 years, WC ≥ 90th percentiles or adult cut-off value if lower.

• Low HDL: For subjects 16 years and older HDL < 40 mg/dL for males and < 50 mg/dL for females. For ages 13–15 years, HDL < 40 mg/dL;

• High TG: TG ≥ 150 mg/dL;

• Hypertension: SBP ≥ 130 mmHg or DBP ≥ 85 mmHg; and

• High Fasting Glucose: ≥ 100 mg/dL.

b Insulin resistance, HOMA-IR > 3.16;

c Hyperinsulinemia, Insulin ≥ 30 μU/ml for Tanner stage 4 and ≥ 20 μU/ml for Tanner stage 5.

IR was identified in 88.5% of obese, 84.6% of overweight and 24.7% of normal weight adolescents (Table II). Similarly, IR and hyperinsulinemia were present in 100% and 73.1% of subjects diagnosed with MS, respectively (Table IV).

Correlation analysis showed that, among subjects of both genders, both BMI and WC were significantly negatively correlated with HDL and significantly positively correlated with TG, SBP, DBP and insulin levels, while %BF was positively associated with TG, DBP, insulin levels and IR (Table V).

**Table V:** Pearson correlation coefficients of metabolic variables under study in subjects of both genders.

Variable	% BF	BMI	WC	HDL	TG	Glucose	SBP	DBP	Insulin	HOMA
% BF	1.000	0.726	0.453	0.049	0.299**	−0.163**	0.011	0.130*	0.465**	0.232**
*p*-value	-	0.000	0.000	0.430	0.000	0.008	0.863	0.035	0.000	0.000
BMI	-	1.000	0.646**	−0.188*	0.289**	−0.111	0.292**	0.225**	0.326**	0.054
*p*-value	-		0.000	0.011	0.000	0.137	0.000	0.002	0.000	0.470
WC (cm)	-	-	1.000	−0.155*	0.344**	0.008	0.343**	0.345**	0.176*	− 0.007
*p*-value	-	-		0.037	0.000	0.912	0.000	0.000	0.017	0.922
HDL (mg/dL)	-	-	-	1.000	−0.336**	−0.029	−0.104	−0.102	−0.204**	−0.161*
*p*-value	-	-	-		0.000	0.693	0.164	0.169	0.006	0.030
TG (mg/dL)	-	-	-	-	1.000	−0.006	0.053	0.168*	0.212**	0.161*
*p*-value	-	-	-	-		0.931	0.480	0.023	0.004	0.030
Glucose (mg/dL)	-	-	-	-	-	1.000	0.024	0.122	0.162*	0.184*
*p*-value	-	-	-	-	-		0.745	0.101	0.029	0.013
SBP (mmHg)	-	-	-	-	-	-	1.000	0.474**	0.020	−0.045
*p*-value	-	-	-	-	-	-		0.000	0.790	0.548
DBP (mmHg)	-	-	-	-	-	-	-	1.000	0.030	0.038
*p*-value	-	-	-	-	-	-	-		0.690	0.612
Insulin (μU/mL)	-	-	-	-	-	-	-	-	1.000	0.411**
*p*-value	-	-	-	-	-	-	-	-		0.000

BF, Body fat; DBP, Diastolic Blood pressure; HDL-C, High Density lipoprotein-Cholesterol; IR, Insulin Resistance; SBP, Systolic blood pressure; TG, Triglycerides; WC, Waist circumference.

* *p* < 0.05;

** *p* < 0.01.

Age- and sex-adjusted linear regression analysis showed that all three adiposity markers (BMI, WC and %BF) were significant predictors of TG, Insulin, SBP and DBP (data not shown). Regression analyses also showed that BMI (OR = 1.255; 95% CI = 1.144−1.377), %BF (OR = 1.157; 95% CI = 1.082−1.239) and WC (OR =1.081; 95% CI = 1.044−1.119) were all significant predictors of MS in the study sample.

## DISCUSSION

This study is the first, to our knowledge, to examine the association between obesity and MS indicators among adolescents from the *Levant* area in the Middle-East. It showed that obesity increases the risk of developing MS among Lebanese adolescents (13–19 years) of Tanner stages 4–5. The prevalence of MS was estimated at 21.2% in obese adolescents using IDF criteria, with obese boys presenting a higher rate as compared to obese girls. Abdominal obesity, reduced HDL-levels and hypertriglyceridemia appeared as the main abnormalities contributing to the syndrome.

Various definitions of paediatric MS have been used in the literature with varied sets of criteria and different cut-off values for each risk factor, making disparities across studies difficult to appraise (Cook et al. 2003; de Ferranti et al. 2004; Weiss et al. 2004; Karachaliou et al. 2008). For comparison purposes, the prevalence of MS in this study was re-calculated using the ATP III definition (Cook et al. 2003) and results were contrasted with those obtained from the region and elsewhere. Findings indicate that the prevalence of MS among obese adolescents in Lebanon (24%) is notably high, exceeding that reported from developed countries such as Italy (16.5%) (Caranti et al. 2008) and Spain (18.0%) (López-Capapé et al. 2006), while being lower than that reported from the US (42%) (Dhuper et al. 2007), the UAE (44%) (Eapen et al. 2010) and Iran (41.9%) (Esmaillzadeh et al. 2006). This discrepancy in MS prevalence among obese adolescents is in line with the assumptions of an ethnic-based association between obesity and MS indicators. Several studies have suggested that the impact of obesity, particularly abdominal obesity on MS abnormalities may vary by ethnic origin (Chateau-Degat et al. 2008). For instance, a study conducted in Norway documented a considerably higher prevalence of MS among overweight and obese children with Middle Eastern origins as compared to their Norwegian counterparts (30.6% and 20.8%, respectively) (Kolsgaard et al. 2008). It is in this context that the use of criteria specific to race or ethnic group for MS in youths is becoming highly warranted (Sumner 2008).

The results of the present study clearly show that the presence of individual cardiometabolic risk factors was high in obese subjects, with 97.1% of obese adolescents presenting with at least one MS abnormality (data not shown). These findings may place obese adolescents at a higher risk for early manifestations of atherosclerosis, as suggested by Whincup et al. (2005), who showed that brachial artery dispensability decreased in obese adolescents as adiposity, IR, diastolic blood pressure and the other criteria of MS increased.

The findings of this study suggest gender disparities in the prevalence of MS, with boys presenting higher prevalence rates as compared to girls. This higher prevalence of MS in boys is in agreement with previous studies conducted in the US (Cook et al. 2003; Weiss et al. 2004), but contradictory to those reported from Iran, another Middle-Eastern country (Esmaillzadeh et al. 2006). One reason behind this gender disparity in our study may be the higher prevalence of severe obesity (BMI > +3 *Z*-scores for sex and age) among boys (8.0%) as compared to girls (5.3%).

In addition to abdominal obesity, which was present in the majority of subjects having the MS, this study showed that low HDL and elevated TG contributed most to MS prevalence (96.2% and 73.1%, respectively). Further analysis of the data by logistic regression showed that the TG/HDL ratio was a strong determinant of MS in the study sample (OR = 4.3, 95% CI = 2.76−6.72). The high prevalence of high TG coupled with low HDL (i.e. a high TG/HDL ratio) among obese subjects has been noted previously as a strong predictor of MS in Venezuelan children (6–12 years) (Quijada et al. 2008), prompting investigators to propose the use of the TG/HDL ratio as a predictive indicator of MS in children and adolescents (Hannon et al. 2006; Dhuper et al. 2007; Li et al. 2008). It is worth noting that, in a national study on the prevalence of MS among Lebanese adults, low HDL appeared as the most prevalent metabolic abnormality in the studied population (49.3%) (Sibai et al. 2008). This is supported by previous findings where reduced HDL levels were reported as the most common metabolic abnormality among adult Arab Americans (Jaber et al. 2004).

In this study, 73.1% of obese adolescents were hyperinsulinemic and 88.5% insulin-resistant, based on HOMA-IR. These findings are similar to those reported from France (Druet et al. 2006) where 71.8% of obese children and adolescents were insulin-resistant, but higher than estimates reported from Bolivia (39.4%) (Caceres et al. 2008), Italy (40.8%) (Valerio et al. 2006), Spain (45.4%) (Tapia Ceballos et al. 2007) and Mexico (51%) (Juarez-Lopez et al. 2010). Hyperinsulinemia serves to compensate for IR to maintain glucose homeostasis (Eyzaguirre and Mericq 2009), as evidenced by the fact that the least common abnormality in the study subjects was elevated fasting blood glucose. Adolescents with hyperinsulinemia and IR are prone to several health problems, including systemic inflammation, endothelial dysfunction, hypertension, polycystic ovary syndrome and early atherosclerosis, all of which can appear during childhood and adolescence in obese individuals (Eyzaguirre and Mericq 2009; Steinberger et al. 2009; Juarez-Lopez et al. 2010). IR has also been suggested to play a pivotal role in the pathogenesis of MS as evidenced by the fact that, in this study, IR was found in all subjects having MS. This central role of IR in the development of MS is corroborated by results from other studies in American, Mexican and Bolivian youth suggesting that MS is more likely to develop in children and adolescents with hyperinsulinemia (Cruz and Goran 2004; Caceres et al. 2008; Shaibi and Goran 2008; Juarez-Lopez et al. 2010). IR has thus been suggested as being a significant predictor of the paediatric MS and was proposed as a diagnostic tool in children and adolescents (Tresaco et al. 2005; Lee et al. 2007).

In this study, regression analyses showed that %BF, BMI and WC were relatively similar in their ability to predict MS and that body fat was not superior to either BMI or WC in predicting metabolic risk in obese adolescents. These findings are in agreement with those reported by Neovius et al. (2009) in Swedish adolescents and Lindsay et al. (2001) in Pima Indian adolescents, thus underscoring the utility of BMI as an adiposity indicator and a predictor of cardiovascular risk factors in this age group. BMI was suggested by Sen et al. (2008) as the most important determinant of MS in a study on obese children and adolescents in Turkey, whereby a one-point increase in BMI *Z*-score resulted in a 2-fold increase in the prevalence of MS (Sen et al. 2008).

## CONCLUSIONS

In conclusion, this study is, to our knowledge, the first in the *Levant* area and in Lebanon to investigate the magnitude of the association between obesity and components of the MS in an adolescent population group. The findings showed that the prevalence of metabolic abnormalities and their clustering into MS increased with obesity and that BMI, WC and %BF were significant predictors of MS in this age group. These findings highlight the importance of early screening for metabolic abnormalities in adolescent obesity and stress the importance of early weight management intervention strategies as the burden of comorbidities may be reversed if health risks are identified and treated early in life (Battista et al. 2009).

The results of this study should be considered in light of the following limitations. Elevated WC was determined, as recommended, according to cut-off points specific for Europids due to the unavailability of WC cut-off values specific for our population. Hyperinsulinemia was also identified based on international cut-off points due to the lack of an upper limit of insulin values for Lebanese population. Finally, we have used fasting glucose and insulin values in the HOMA equation to estimate IR, rather than other more accurate techniques such as the euglycemic clamp.
